# Biogeographic discordance of molecular phylogenetic and phenotypic variation in a continental archipelago radiation of land snails

**DOI:** 10.1186/1471-2148-14-2

**Published:** 2014-01-07

**Authors:** Sean Stankowski, Michael S Johnson

**Affiliations:** 1 School of Animal Biology (M092), University of Western Australia, Crawley, Western Australia 6009, Australia; 2 Institute of Ecology and Evolution, University of Oregon, Eugene, Oregon 97403-5289, USA

**Keywords:** Island biogeography, Parallel evolution, Phylogeography, *Rhagada*

## Abstract

**Background:**

In island archipelagos, where islands have experienced repeated periods of fragmentation and connection through cyclic changes in sea level, complex among-island distributions might reflect historical distributional changes or local evolution. We test the relative importance of these mechanisms in an endemic radiation of *Rhagada* land snails in the Dampier Archipelago, a continental archipelago off the coast of Western Australia, where ten morphospecies have complex, overlapping distributions.

**Results:**

We obtained partial mtDNA sequence (COI) for 1015 snails collected from 213 locations across 30 Islands, and used Bayesian phylogenetic analysis and Analysis of Molecular Variance (AMOVA) to determine whether geography or the morphological taxonomy best explains the pattern of molecular evolution. Rather than forming distinct monophyletic groups, as would be expected if they had single, independent origins, all of the widely distributed morphospecies were polyphyletic, distributed among several well-supported clades, each of which included several morphospecies. Each mitochondrial clade had a clear, cohesive geographic distribution, together forming a series of parapatric replacements separated by narrow contact zones. AMOVA revealed further incongruence between mtDNA diversity and morphological variation within clades, as the taxonomic hypothesis always explained a low or non-significant proportion of the molecular variation. In contrast, the pattern of mtDNA evolution closely reflected contemporary and historical marine barriers.

**Conclusions:**

Despite opportunities for distributional changes during periods when the islands were connected, there is no evidence that dispersal has contributed to the geographic variation of shell form at the broad scale. Based on an estimate of dispersal made previously for *Rhagada*, we conclude that the periods of connection have been too short in duration to allow for extensive overland dispersal or deep mitochondrial introgression. The result is a sharp and resilient phylogeographic pattern. The distribution of morphotypes among clades and distant islands is explained most simply by their parallel evolution.

## Background

Biogeographic patterns reflect historical distributional changes and local evolution [1,2]. In island archipelagos, where marine barriers restrict dispersal, the relative importance of these mechanisms depends on historical patterns of connection of islands to one another and to the mainland, and the dispersal capabilities of the organism in question [3,4]. For example, in oceanic archipelagos, where islands have always been isolated, there is limited opportunity for range expansion and gene flow, which tends to favour the evolution of island endemics [5]. Continental archipelagos, on the other hand, have usually experienced repeated periods of fragmentation and connection through cyclic changes in sea level, which tend not to favour local diversification. In many cases, periods of connection have been much longer than the periods of isolation, enabling complex among-island distributions to develop through range expansion and vicariance [3,4].

**Figure 1:**
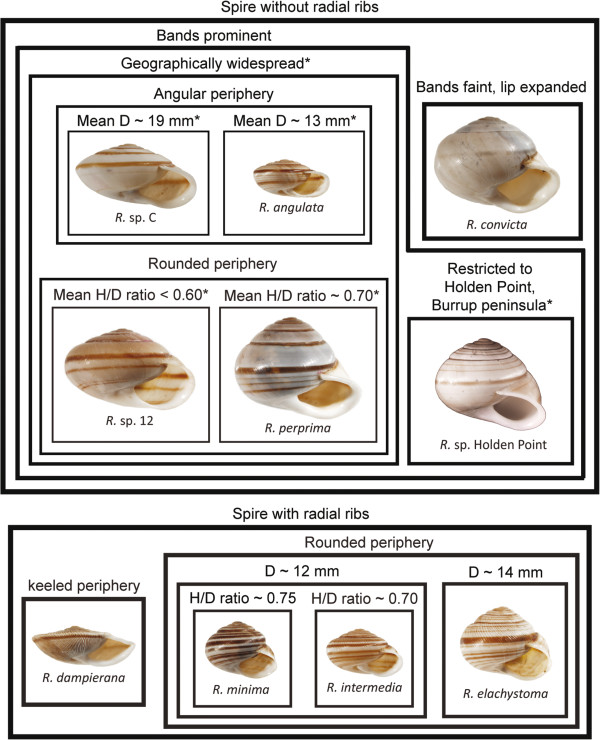
**Described and putative morphospecies of *Rhagada *from the Dampier Arhcipelago.** Boxes at the same hierachical level represent alternative states of a common morphological character, descibed above each box, so that species in the same box share the same character state. Characters marked with an asterisk have been added to incorperate the undescribed forms into the existing taxonomy (see Methods). D = shell diameter, H = shell height. Shells are shown at a common scale.

Despite its small area and continental origin, the Dampier Archipelago in Western Australia has supported local evolution, where land snails from the genus *Rhagada* have undergone extensive morphological diversification [6,7]. Of the 31 described Western Australian species of *Rhagada*[8-10]*,* which occupy approximately 200,000 km^2^, seven are found in the 250 km^2^ Dampier Archipelago. Six of those species and a further three undescribed forms are endemic to the islands [7,8]. Probably the most striking feature of *Rhagada* in the Dampier Archipelago is its extreme variation in shell size, shape, sculpture and pattern of banding, which spans the entire range of the genus, and is the primary basis for the current taxonomy [8] (Figure 1). Recent phylogenetic analysis of the Western Australian species of *Rhagada* revealed three clades in the Archipelago [7]: Clades C and D are endemic to the islands, while Clade A, a primarily mainland lineage, has a marginal distribution in the Dampier Archipelago. Five of the described species and the three undescribed island forms are confined to the endemic Clade D, suggesting that they have evolved as a local radiation [7]. However, that phylogenetic study did not provide a thorough test of evolutionary relationships within the Dampier Archipelago, because each species was represented by specimens from a single location [7].

In the context of the genus *Rhagada*, the Dampier Archipelago is remarkable not only for the extreme morphological diversity in a relatively small area, but also because it is the only known location where species of *Rhagada* have overlapping geographic distributions [8]. On the adjacent mainland, species have allopatric or parapatric distributions spanning 150 km or more [8]. In contrast, five of the ten Dampier Archipelago *Rhagada* have overlapping distributions that encompass several, often distant islands, with up to five species recorded on one island. Given the extent of the morphological diversification within the group, and repeated connection and fragmentation of the islands during cyclic changes in sea level, during which the coastline shifted more than 100 km [7], contrasting scenarios might explain the complex distributions of the morphospecies. First, in agreement with the taxonomy, each species may have evolved once, and, through overwater dispersal or vicariance, came to occupy several islands. A prediction from this single-evolution hypothesis is that the existing taxonomy should be the best predictor of the pattern of molecular subdivision within the group. An alternative explanation is that the divergent shell morphologies, considered to be characteristic of each species, have evolved independently in more than one location, in which case, the pattern of molecular subdivision should reflect geography, not the current taxonomy. A previous study of populations on a single island in the Dampier Archipelago, Rosemary Island, favoured the latter explanation, as specimens of five of the described species share a monophyletic ancestry within preliminary samples collected from other islands, suggesting that the diversity evolved locally [11].

The present study aims to differentiate between these alternative hypotheses, based on comprehensive population samples collected from 30 islands, mitochondrial DNA sequences, and the existing taxonomy. In addition to assessing the relative roles that local evolution and dispersal have played in establishment of the contemporary biogeographic pattern, the study provides a thorough test of the existing taxonomic hypothesis for the group.

## Methods

### Study location and samples

The Dampier Archipelago (20° 33′ S, 116° 36′ E) comprises 46 islands (0.1 to 170 km^2^) that lie within a 50-km radius of the coastal town of Dampier in Western Australia. The islands, including the Burrup Peninsula, which is now connected to the mainland by a man-made causeway, have been in their current form since rising sea levels flooded what were low-lying coastal plains 6000 to 8000 years ago. During the 120,000 years prior to that, the islands were connected as part of the Australian mainland, and were located more than 100 km inland from the historical coastline. There have been 6 to 8 cycles of fragmentation and connection in the last million years, which is the period of time that *Rhagada* are thought to have inhabited the region [6]. However, for most of that time (~ 900,000 years) the area was connected, with each bout of flooding lasting less than 10,000 years. There have also been a few brief periods in which sea levels were higher than at present, most recently during the Eemian Historical Highstand, approximately 130,000 years ago, when sea levels were elevated by 5 to 10 m for roughly 6000 to 8000 years [12,13]. As a result, some of the current islands were fragmented into groups of smaller islands.

**Figure 2:**
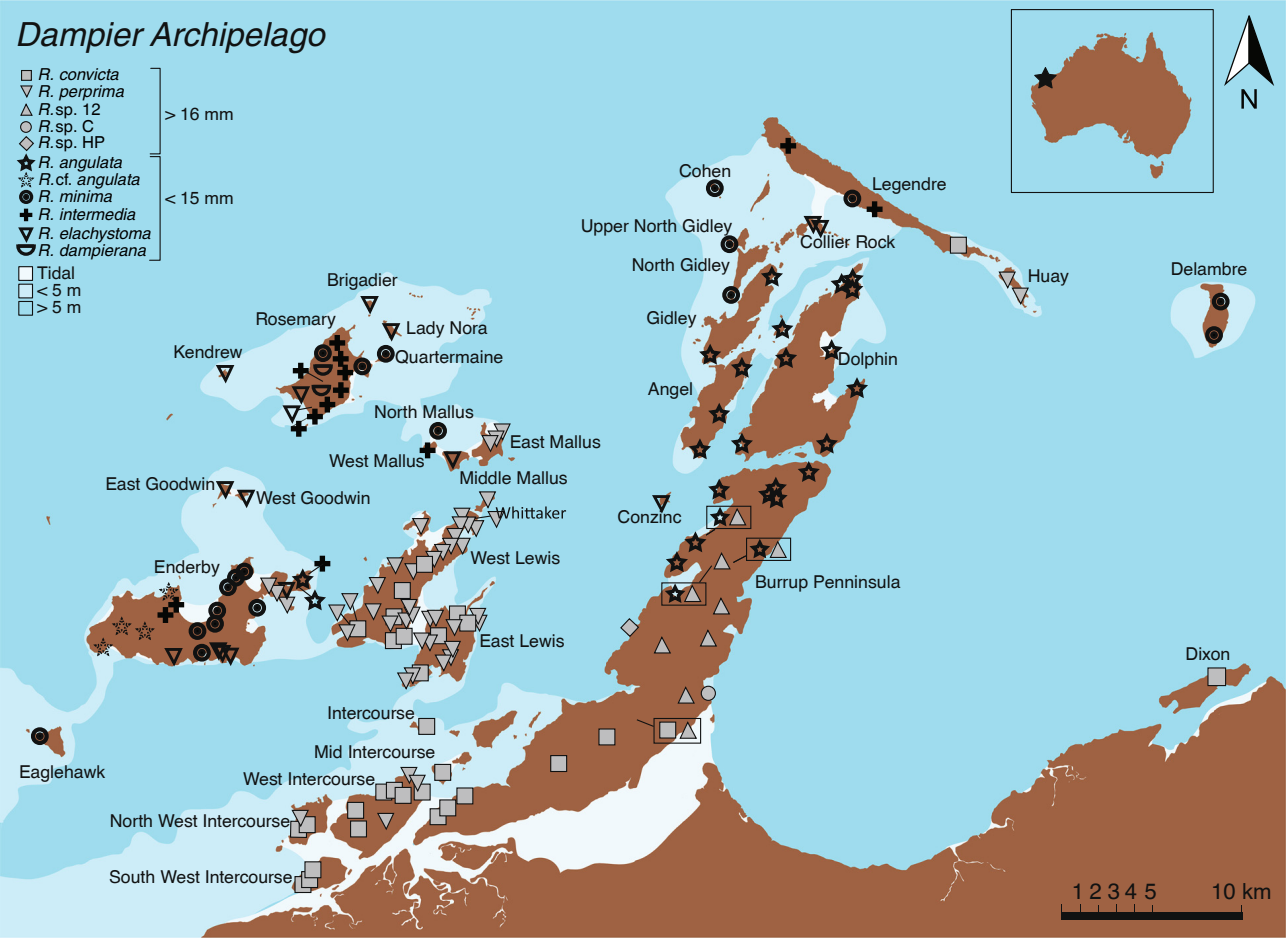
**Sampling locations in the Dampier Archipelago coded by morphospecies.** Black and grey symbols represent small (mean diameter < 15 mm) and large (mean diameter >16 mm) species, respectively. Sites with more than one species are represented by multiple symbols enclosed by a box. Names refer to islands. Different shades of blue illustrate variation in ocean depth.

*Rhagada* snails were collected from 213 locations across 30 islands between 2008 and 2010 (Figure 2). At each site, we collected a maximum of six of each morphospecies that was present. Only adults were collected, distinguished from juveniles by the presence of a reflected lip at the shell aperture, which marks the completion of shell growth [14]. In almost all cases, each sample site had a 15-meter radius, which is an area smaller than the genetic neighbourhood estimated for the mainland congener *Rhagada capensis*[12]. Thus it is reasonable to assume that each sample was collected from a single panmictic unit.

Upon return to the laboratory, snails were identified using the taxonomic key of Solem [8] which is based on variation in shell morphology. The taxonomy was based on very limited material, mostly collections of empty shells lodged with the Western Australian Museum [8]. Five of the species were described primarily from their shells, but also with a qualitative assessment of reproductive anatomy. The remaining two species, *R. minima* and *R. elachystoma,* were described entirely from empty shells. A recent study [11], including material from five of the island endemic species, was unable to discriminate the divergent shell morphologies based on quantitative analysis of the reproductive system. Here, we focus only on the variation in shell form, and refer to the described forms as ‘morphospecies’.

Assigning samples to the described species serves two purposes. First, the key provided a convenient method for sorting samples with similar phenotypes into groups that span the range of variation in shell size, shape, sculpture and banding pattern (Figure 1). We used these groups as the basis for testing how distributional changes and local evolution have contributed to the current biogeography. Second, we were able to conduct a concurrent test of the taxonomic hypothesis. Of the 7 described species that are found in the Dampier Archipelago, 6 are endemic to the islands [7,8], so the material examined here is a comprehensive sampling over their distributions. The seventh, *R. convicta*, is the most widespread species in the genus, and the only one that occupies the Dampier Archipelago and mainland. It is genetically complex across its mainland distribution [7], but the current study only includes island populations.

Aspects of the taxonomic key are open to some interpretation, either because they are subjective (e.g. faint versus bright bands, or expanded versus narrow aperture), or because values are approximate. For example, populations of *R. intermedia* have a mean height/diameter ratio of about 0.70 and, on that basis, can be distinguished from *R. minima*, which has a mean H/D ratio of about 0.75. In this, and other similar situations, we assigned specimens to the species with the closest value, which is reasonable given the ranges of intraspecific variation described for most shell traits [8].

In addition to the seven described species, three undescribed forms have been collected on the Burrup Peninsula, which was not included in Solem’s [8] taxonomic revision. Given that there is morphological and phylogenetic evidence that these forms are distinct from those already described [7], they were incorporated into a revised version of the identification key (Figure 1), based on shell measurements made by Johnson *et al.*[7] and characters that have been used to distinguish other species of *Rhagada*[8]. One undescribed form, *R*. sp. Holden Point, is morphologically cryptic, but has been recognized as locally distinct based on mtDNA sequence, and is geographically restricted to Holden Point on the west side of the Burrup Peninsula. Because its main diagnostic character is its geographic position, only specimens from Holden Point were assigned to this taxon. Following identification, aestivating snails were exposed to moisture and frozen at −80°C prior to DNA extraction.

### Molecular methods and analysis

Total DNA was extracted from up to six individuals per morphospecies per site using Qiagen (Hilden, Germany) DNeasy blood-tissue kits, or the glass fiber plate method of Ivanova *et al.*[15]. Previous phylogenetic studies of this group have collectively examined sequences from two nuclear (ITSII and 18 s) and two mitochondrial genes (16 s and COI) [7,11]. Neither of the nuclear genes were phylogenetically informative in the Dampier Archipelago, due to the close evolutionary relationships. Here, A 633 bp fragment of COI, the mitochondrial gene with the highest phylogenetic resolution, was amplified using the forward (L1490-Alb) and reverse (H2198-Alb) primers of Gittenberger *et al*. [16], according to Stankowski [11], except that the annealing temperature was adjusted between 40°C and 51°C, depending upon the population. Sequences were edited and aligned with Sequencher v.4.6 and deposited in GenBank [accession numbers KF151868 - KF152882].

Phylogenetic analysis was conducted in MrBayes v. 3.2.0 [17]. The best-fit substitution model (HKY + G + I) was determined using JModeltest [18], based on Akaike (AIC) and Bayesian (BIC) information criteria. Five replicate analyses were conducted, each consisting of 4 × 10^7^ generations with sampling every 1 × 10^3^ generations, 2 parallel runs, 4 Markov chains (3 heated, 1 cold) and a burn-in fraction of 0.25. Convergence within runs was assessed based on the average standard deviation of split frequencies, and among runs using the MCMC convergence diagnostic program AWTY [19]. A fifty-percent majority rule consensus phylogram was constructed from post burn-in genealogies. As the relationships between the three major clades in the Dampier Archipelago were unresolved in a previous phylogenetic analysis of the genus [7], the phylogeny was drawn as an unrooted network in FigTree v. 1.3.1 [20].

Arlequin v. 3.5.1 [21] was used to calculate model-based genetic distances within (*Pi*_*X,Y*_) and among (*Pi*_*XY*_) each of the major clades revealed by the phylogenetic analysis, and then the corrected average among-clade pairwise distances were calculated as *Pi*_*XY*_–(*Pi*_*X*_ + *Pi*_*Y*_)/2, which is the average of distances between two groups, minus the average distance between individuals within those groups. As the HKY model is not implemented in Arlequin, distances were estimated using the most similar substitution model, the Tamura-Nei model, with Gamma distributed rates (TrN + G). Hierarchical Analysis of Molecular Variance (AMOVA) of the morphologically diverse endemic Clade D was then conducted in Arlequin to determine whether geography or the taxonomy better explained the pattern of sequence divergence. Two separate analyses were conducted, both using model-based distances. First, variation was partitioned (i) among islands, (ii) among sample sites within islands and (iii) within sample sites. Second, variation was portioned (i) among species, (ii) among sample sites within species and (iii) within sample sites. The significance of the variation at each level was determined from 10,000 random permutations of the data.

## Results

### Distributions of the morphospecies

All individuals could be identified as one of the previously recognized morphospecies (Figure 2), except for some samples from the western end of Enderby Island. These were similar in form to *R. angulata* in most respects, having a mean diameter of approximately 13 mm, prominent bands and a sharply angular body whorl. However, all these specimens had sculptured shells, whereas *R. angulata* is described as a smooth-shelled form. This previously unknown form will be provisionally referred to as ’*R.* cf. *angulata*’.

At each of the 213 sites, only a single morphospecies was found, except at four locations on the Burrup Peninsula, where pairs of morphospecies were collected in sympatry. In the south, *R. convicta* was collected with *R*. sp. 12 at a single location where their distributions abut. The other three sites were from the northern section of the Burrup, where *R*. sp. 12 was collected in sympatry with *R. angulata*.

Five morphospecies were restricted to single islands: *R.* cf. *angulata*, from the western portion of Enderby Island; the three undescribed, smooth-shelled forms (*R*. sp. C, *R*. sp. 12 and *R*. sp. Holden Point) restricted to the central area of the Burrup Peninsula; and the sculptured, keeled-flat species, *R. dampierana*, found only on Rosemary Island. The other six morphospecies had complex, overlapping distributions that spanned several, often distant islands (Figure 2). *R. convicta*, the only morphospecies that occupies the Dampier Archipelago and the mainland, was collected on ten islands, though it was previously known in this study area from only the Burrup Peninsula. Populations of *R. convicta* were generally on islands close to the mainland, including Dixon Island at the far east of the Archipelago and Intercourse and Lewis Islands to the west. However, due to outlying samples collected on Legendre Island and Dixon Island, *R. convicta* had the second largest distribution in the group. The distribution of *R. convicta* showed considerable overlap with that of *R. perprima*, another smooth-shelled, large morphospecies, but distinguished by the presence of prominent shell banding. The main distribution of *R. perprima* included six islands in the west of the Archipelago: Enderby and West Lewis Islands, from which it was previously recorded, and East Lewis, East Mallus, and Southwest and West Intercourse Islands. However, two outlying samples were also collected on Hauy Island, located approximately 35 km to the northeast.

*R. angulata*, distinguished from other smooth-shelled, prominently banded morphospecies by its smaller size, was collected on five islands, spanning approximately 50 km. The main distribution included the northern end of the Burrup Peninsula, and Dolphin, Angel, and Gidley Islands, directly to the north. It was also found on the eastern end of Enderby Island, approximately 20 km to the west, and the similar but sculptured *R.* cf. *angulata* was collected on the western end of Enderby Island.

The three small-shelled, sculptured morphospecies *R. minima*, *R. intermedia* and *R. elachystoma* had broadly overlapping distributions, occupying the islands that were most distant from the coast. *Rhagada minima*, distinguished from *R. elachystoma* based on shell diameter (12 mm and 14 mm respectively) and from *R. intermedia* based on the ratio of shell height to the diameter (0.75 and 0.70, respectively), had the broadest geographic distribution of any morphospecies. Populations of *R. minima* were collected on ten islands, from Eaglehawk Island in the far west to Delambre Island in the east, spanning more than 60 km. Previously recorded on Enderby, Conzinc and Rosemary Islands, where it was also collected in the present study, the known distribution of *R. minima* was expanded to include Quartermaine, North Mallus, Legendre, Cohen, and North and Upper North Gidley Islands. *R. elachystoma*, previously known only from Rosemary and Kendrew Islands, which are separated by less than 5 km, was collected on nine islands, all west of the Burrup Peninsula. With the exception of Rosemary and Enderby Islands, all were very small, less than 1.5 km wide. *R. intermedia* was collected from four islands (Legendre, Rosemary, Enderby and West Mallus), spanning approximately 40 km.

Complex distributions were also observed within islands. Except for the smallest islands, many of which were sampled at only one location, and a few of the larger ones, each island was inhabited by several morphospecies. Richness was highest on Enderby Island, with eight morphospecies, including the new form *R.* cf. *angulata*. Rather than being distributed as a series of allopatric or parapatric replacements, morphospecies were interspersed in complex, overlapping distributions. Similar distributions were observed on several other islands, including Rosemary, Legendre, West Intercourse and East and West Lewis Islands.

### Molecular variation and phylogeographic patterns

**Figure 3:**
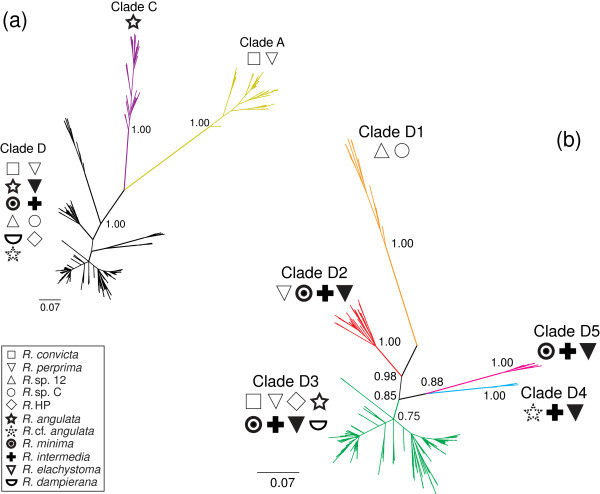
**Phylogenetic relationships. (a)** Unrooted fifty-percent majority rule Bayesian consensus phylogram constructed from a 633 bp fragment of the mitochondrial gene Cytochrome Oxidase I (*n* = 1015), coded to show the three clades (A, C and D) recognized by Johnson *et al*., (2012) [7]. **(b)** Inset showing the five subclades recognized within the island endemic clade D. Taxon names show the phylogenetic distribution of the described/putative species, excluding sites with mixed ancestry (see Figure 4). Values represent the posterior probabilities for each node.

Haplotype diversity was high, with 640 unique haplotypes recovered from 1015 snails. Of the 633 bp, 266 sites were variable, and 248 of those were phylogenetically informative. The phylogenetic analysis revealed the three major clades already known to inhabit the Dampier Archipelago, Clades A, C and D (Figure 3). These clades were separated by 14.2% to 19.1% sequence divergence, and supported by posterior probabilities of 1.00.

Clade D, the morphologically diverse island endemic clade, was further subdivided into five shallower clades, D1 to D5, with bootstrap support between 0.75 and 1.00. Corrected model-based distances between the clade D sub-clades ranged from 2.9% between clades D3 and D4 to 10.1% between clades D1 and D5 (Table 1).

**Table 1:** Mean sequence divergence For COI within and among clades, calculated from the number of differences

	**A**	**C**	**D1**	**D2**	**D3**	**D4**	**D5**
A	**0.045**	0.191	0.194	0.164	0.175	0.177	0.180
C	0.139	**0.059**	0.178	0.142	0.143	0.150	0.160
D1	0.153	0.131	**0.036**	0.104	0.110	0.112	0.126
D2	0.131	0.103	0.076	**0.021**	0.059	0.062	0.093
D3	0.138	0.099	0.077	0.034	**0.030**	0.066	0.084
D4	0.132	0.098	0.072	0.030	0.029	**0.044**	0.080
D5	0.150	0.124	0.101	0.075	0.062	0.051	**0.015**

Diagonal elements: mean proportion of within-clade pairwise differences. Above the diagonal: mean between-clade pairwise differences. Below the diagonal: mean between-clade corrected pairwise difference.

**Figure 4:**
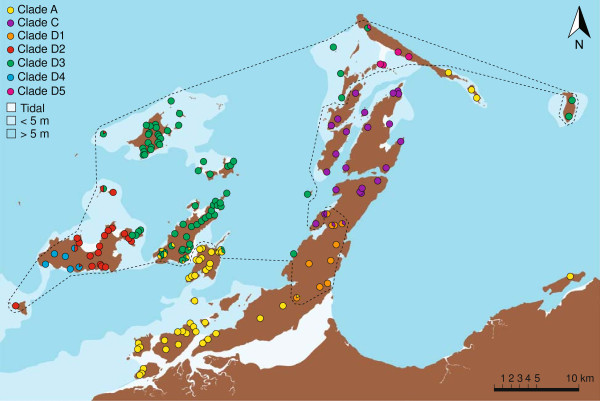
**Phylogeographic patterns in the Dampier Archipelgo.** Each sample site is represented by a pie chart divided to show the proportion of individuals that correspond to each mitochondrial clade. The dashed line envelopes the distribution of the locally evolved Clade D. Different shades of blue illustrate variation in ocean depth.

In contrast to the complex, overlapping distributions of morphospecies, the mitochondrial clades had clear, cohesive geographic distributions (Figure 4). Of the three main clades, A, C and D, clade D had the broadest distribution in the archipelago, including 25 islands. Clade C was the most restricted, found on four islands. With the exception of a few outlying populations, each clade generally occupied a group of neighbouring islands, together forming a series of parapatric replacements. Zones of contact, consisting of populations of mixed ancestry, were observed between clades A and D on East and West Lewis Islands and between clades A and D and clades C and D on the Burrup Peninsula. The zones of contact were all much narrower than the main distributions of the associated clades.

The same pattern was observed for the subclades of clade D. Apart from clades D1 and D4, which were restricted to single islands, each clade occupied a group of neighbouring islands, and together formed a series of parapatric replacements. Zones of contact were observed between clades D2 and D3 on West Goodwin and Kendrew Islands, D2 and D4 on Enderby Island and D3 and D5 on Legendre Island.

### Associations between molecular variation and taxonomy

There was incongruence between the taxonomic and phylogenetic hypotheses, even between the three main clades (Figure 3). Clade A contained individuals assigned to two morphospecies, *R. convicta* and *R. perprima*. Individuals assigned to both of these morphospecies were also distributed within the morphologically diverse clade D, which contained all 11 morphospecies. Similarly, while, individuals from clade C were all identified as *R. angulata*, other specimens of *R. angulata*, collected 20 km west of its main distribution, fell within clade D.

More extensive conflict between the taxonomic and phylogenetic hypotheses was observed within clade D, where four of the morphospecies were found in more than one subclade. *R. intermedia* and *R. elacystoma* were distributed among four clades, D2, D3, D4 and D5, while *R. minima* was distributed among three, D2, D3 and D5, and *R. perprima* among two, D2 and D3. The only morphospecies restricted to single clades were those found only on single islands: *R. dampierana*; the three undescribed forms from the Burrup (*R.* sp Holden Point*, R.* sp. 12 and *R.* sp. C); and *R.* cf. *angulata. Rhagada* sp 12. and *R*. sp. C formed clade D1, and were the only two morphospecies in that clade. Clade D3 was richest in morphological diversity, with eight morphospecies, while clades D4, D5, and D2 were associated with three, three and four morphospecies, respectively.

Analysis of Molecular Variance quantified the disparity between molecular variation and the taxonomic hypothesis within the Clade D subclades (Table 2). In the taxonomic analyses, which partitioned variation among morphospecies, among sites within morphospecies and within sites, the taxonomy never explained the largest proportion of variation, ranging from 11.9% to 36.4% (mean 21.7% ± SD 9.7), and in four clades was not statistically significant. For three clades, the majority of variation was distributed among sites within morphospecies (mean 50.9% ± SD 7.4), while for the other two it was within sites (39% for 61% Clades D3 and D5, respectively). In the geographic analyses, which partitioned variation among islands, among sites within islands and within sites, islands explained the most variation in clade D3 (61%), which was the most geographically widespread clade D subclade, spanning 22 islands. For the other two clades that occupied more than one island, D2 and D5, the majority of variation was distributed among sites within islands and within sites, respectively.

**Table 2:** Results of Analysis of Molecular Variance of COI sequence divergence within each subclade of the Dampier Archipelago endemic Clade D

**Clade**	**Grouping**	**Level of comparison**	**df**	**ss**	**Var. Comp.**	**%**	***p***
D1	Taxonomy	Among species	1	55.35	1.56	11.88	0.2690
		**Among sites, within species**	**9**	**293.69**	**7.66**	**58.39**	**< 0.0001**
		Within sites	32	124.88	3.90	3.90	< 0.0001
		Total	42	473.93	13.13		
	Geography	Among islands	-	-	-	-	-
		**Among sites**	**10**	**349.04**	**8.02**	**67.27**	**< 0.0001**
		Within sites	32	124.88	3.90	32.73	-
		Total	42	473.93	11.92		-
D2	Taxonomy	Among species	4	115.99	0.95	14.39	0.0873
		**Among sites, within species**	**13**	**207.33**	**2.89**	**43.59**	**< 0.0001**
		Within sites	64	178.06	2.78	42.02	< 0.0001
		Total	81	501.38	6.62		
	Geography	Among islands	4	115.63	1.96	24.37	0.1073
		Among sites, within islands	14	244.04	3.01	37.40	< 0.0001
		**Within sites**	**69**	**212.15**	**3.07**	**38.23**	**< 0.0001**
		Total	87	571.82	8.04		
D3	Taxonomy	Among species	6	1509.62	3.83	36.36	< 0.0001
		Among sites, within species	97	1485.62	2.61	24.75	< 0.0001
		**Within sites**	**349**	**1429.06**	**4.09**	**38.88**	**< 0.0001**
		Total	452	4424.29	10.53		< 0.0001
	Geography	**Among islands**	**19**	**2654.83**	**7.18**	**61.23**	**< 0.0001**
		Among sites, within islands	106	673.71	0.63	5.36	< 0.0001
		Within sites	394	1543.09	3.92	33.42	< 0.0001
		Total	519	4871.63	11.72		
D4	Taxonomy	Among species	2	21.26	6.29	25.64	0.7036
		**Among sites, within species**	**2**	**117.32**	**11.73**	**50.69**	**< 0.0001**
		Within sites	12	70.53	5.88	23.67	< 0.0001
		Total	16	209.12	23.90		
	Geography	Among islands	-	-	-	-	-
		**Among sites**	**4**	**149.57**	**9.90**	**61.42**	**0.0030**
		Within sites	12	74.67	6.22	38.58	-
		Total	16	224.24	16.13		
D5	Taxonomy	Among species	2	36.80	1.08	20.31	0.0687
		Among sites, within species	2	15.34	0.96	18.10	0.0630
		**Within sites**	**22**	**72.14**	**3.28**	**61.59**	**< 0.0001**
		Total	26	124.29	5.32		
	Geography	Among islands	1	17.90	0.37	7.04	0.1967
		Among sites, within islands	3	34.25	1.62	30.74	0.0011
		**Within sites**	**22**	**72.14**	**3.28**	**62.22**	**< 0.0001**
		Total	26	124.29	5.27		

For each analysis, the largest component of variance is highlighted in bold face.

## Discussion and conclusion

The primary aim of this investigation was to understand the roles that distributional changes and local evolution have played in establishing the complex biogeographic patterns in the Dampier Archipelago, and, at the same time, evaluate the existing morphological taxonomy for *Rhagada*. According to the current taxonomic hypothesis, each shell morphotype is characteristic of a single, reproductively isolated species [8], implying that the complex distributions originated through fragmentation of broader historical distributions or overwater dispersal. The results of this study do not support that view. Rather than forming distinct monophyletic groups in the phylogenetic analysis, as would be expected if they were good species with single, independent origins, all the widely distributed morphotypes are distributed among several divergent mitochondrial clades, each of which includes several of the morphospecies. The disconnection is extensive within the morphologically diverse island endemic Clade D, but also occurs among the major *Rhagada* lineages revealed by Johnson *et al*. [7].

Based on these results, the incongruence between morphological and molecular phylogenetic variation may be explained in three ways, which are not mutually exclusive. These include (i) introgressive hybridization, (ii) retention of ancestral polymorphism and (iii) the parallel evolution of variation among clades. These data suggest a minor role for introgressive hybridization at the broad scale. This is most clear for the introgression of mtDNA haplotypes, with the exception of populations of mixed phylogenetic ancestry, which we excluded from our discordance analyses. We arrive at this conclusion by inferring the geographic scale of introgression during periods when the islands were connected. Upon secondary contact, neutral markers diffuse into the opposing gene pool leading to the formation of a cline with a width of *w* = *σ*√*T*/0.35, where *T* is the time since contact and *σ* is a time-calibrated estimate of dispersal [22]. The model assumes that there is no selection against hybrids, or *w* would be overestimated. Stankowski [23] estimated dispersal in *Rhagada* from Rosemary Island as *σ* = 9.45 m/generation (95% ci = 7.84 – 12.82 m. gen.). In the Dampier Archipelago, periods of connection lasted a maximum of 120,000 years during the time that *Rhagada* are thought to have inhabited the region [6]. Assuming that it takes five years for *Rhagada* to reach sexual maturity, as estimated for *R. capensis*[14], this gives *T* = 24,000 generations of contact. Substituting these values into the equation, we obtain a cline width of *w* = 2.47 km (95% ci = 2.05 – 3.36), which equates to 1.24 km (95% ci 1.03 – 1.68 km) of introgression into each side (i.e., diffusion in one direction = 0.5*w*). These estimates, compatible with the dimensions of extant contact zones observed between the clades, suggest that introgression during periods of connection would have been restricted to very narrow regions located in areas that are now ocean floor, having little impact on the core structure of populations. While suggesting that the broad-scale discordance between phylogeny and morphology cannot be a result of introgression, the low rate of dispersal of *Rhagada* does explain how the striking phylogeographic pattern has persisted, given that the islands have been connected for more than 900,000 of the past million years [13].

Hybridization may also generate incongruence through the introgression of selectively favoured alleles, such as those coding for the shell phenotype, into an alternative phylogenetic background. In this case, the speed of introgression is not limited to diffusion, because selection can rapidly drive an allele to fixation in an area where it is favored. Estimating the maximum speed of the spread of an advantageous allele or quantitative trait is complicated, and requires parameter estimates that we do not have. However, we exclude it as a main cause of the broad biogeographic disparity, because transitions between clade boundaries are typically associated with changes in the shell morphotype. While introgression may have played an important role in specific cases, an extremely complex, highly unlikely set of scenarios would be required to explain the broad incongruence between phylogenetic and morphological variation.

The geographic scale and complex pattern of the discordance implicates the remaining explanations. At the broadest phylogenetic and spatial scales, parallel evolution is almost certainly responsible. Parallel or convergent evolution of shell form is relatively common, having been documented in several closely related lineages of land snails [24-26]. In *Rhagada*, the clearest examples include the independent evolution of the *R. angulata* morphotype in Clades C and D, keeled shells in clades D1 and D3, and angular peripheries in clades A, D1 and D3. While parallel evolution may also explain the sharing of morphological variation among the locally evolved Clade D subclades D2, D3, D4 and D5, much of the morphological variation may have been present in the common ancestor, and retained to varying extents in each clade.

Regardless of how it has become distributed among the clades, the variation is probably of adaptive significance. The geographic cohesion of populations of similar-sized shells and the sharp spatial transition between exclusively large- and small-shelled populations suggest that divergent selection favours these alternative forms in different areas. Moreover, small and large shells are almost exclusively associated with sculptured and smooth shell surfaces, respectively, suggesting that there may be coadaptation between traits. For these examples, there is no evidence of what the selective agent may be. Shell shape, on the other hand, appears to be associated with contrasting habitats. On Rosemary Island, where there is striking geographic variation in shell shape spanning the range of variation in the genus, there is a clear, repeated association between rocky hills and the keeled-flat form (previously described as *R. dampierana*), and strong evidence that divergent ecological selection is driving speciation in the face of gene flow [23]. This may also be the case for populations in Clade D1 on the Burrup Peninsula, where the flattened, occasionally keeled *R*. sp. C is restricted to a similar rocky hill, while taller-shelled populations of *R*. sp. 12 occupy sandy, low-lying habitats [7]. Likewise, the low-spired angular forms *R. angulata* and *R.* cf. *angulata*, collected on several islands, were found exclusively in rocky habitats (Stankowski, personal observations). The replication of these contrasting environments throughout the archipelago provides an excellent opportunity to test the generality of this association at broader scale, both within and among clades.

While extrinsic effects may explain the evolution of similar phenotypes among the islands, they are unlikely to account for the different levels of morphological variation observed among the major lineages. Consistent with earlier analyses [7], the vast majority of the morphological variation is observed in one of the three major clades that inhabit the Dampier Archipelago, the island endemic Clade D, including the *R. angulata* morphotype, which was previously thought to be restricted to the monotypic Clade C. Clade D includes the full range of variation in shape, sculpture and pattern of banding observed in the genus, and nearly the full range of variation in size, contrasting with the morphological conservatism observed within other lineages. This observation is explained most simply by variation in the intrinsic evolvability among lineages. Variation in evolvability has been cited to explain why some lineages diversify within a given setting and others do not [27-29]. In *Rhagada*, including additional mainland lineages not represented in the Dampier Archipelago, only Clade D exhibits the exceptional diversity of shell form [7], suggesting that it too has a special capacity for morphological evolution.

While far exceeding the levels of variation observed within many species of land snails, and even within entire genera, the morphological diversity within clades D1 to D5 almost certainly reflects striking geographic variation. As a measure of the independence of morphological and molecular evolution, the morphological taxonomy explained only a small, generally non-significant proportion (12 to 36%) of the within-clade molecular variation. For example, in Clade D3, which includes, specimens from eight of the ten morphospecies, spanning the full range of shell shape and sculpture in the entire genus, and nearly the full range of size, the average corrected COI sequence divergence is 3%, which is half the threshold of 6% observed between anatomically distinct species in a study of *Amplirhagada*, another northwestern Australian camaenid genus [30]*.* More of that variation is distributed within sample sites than among morphospecies, and in the morphologically most diverse Clade D3, current marine barriers explain 61% of the COI variation. Direct support for the hypothesis of reproductive continuity within clades also comes from detailed studies on Rosemary Island [23], all from Clade D3, where nuclear microsatellite DNA provides clear evidence for gene flow between specimens that key to four of the described morphospecies, with a maximum percentage sequence divergence of COI of 4.4% (mean 1.24 ± SD 0.78).

There is, however, good evidence that speciation is complete or nearly complete, between the three major lineages recognized by Johnson *et al.*[7]. The two clearest examples are on the Burrup Peninsula, where the large, rounded form *R*. sp. 12 from Clade D1 was collected in microsympatry in the north with the small, angular form *R. angulata* from Clade C, and in the south with *R. convicta* from Clade A. No intermediate shells or phylogenetic incongruence were observed, and the association between molecular and morphological divergence is complete, with mean corrected COI divergence of 13.1 to 15.3%. The reproductive relationships between Clade A and Clade D3, which are separated by a similar level of divergence (13.8%), are less clear in the contact zone that cuts through East and West Lewis Islands. The two clades are represented respectively by the widespread *R. convicta* and the island endemic *R. perprima*, which are morphologically extremely similar, differing mainly in the intensity of shell banding [8]. There is some incongruence between COI clades and intensity of shell banding in the contact zone, but it is not clear whether this is due to hybridization or to local adaptation. Indeed, other examples of intensely banded individuals with *R. convicta* mtDNA were found on Hauy Island, nearly 30 km from the nearest *R. perprima*. Banded populations of *R. convicta* have also been collected from a few mainland sites (Z.R. Hamilton unpublished), suggesting that the intensity of shell banding is too labile to be a reliable taxonomic trait. More thorough analyses, including an examination of nuclear genes and a quantitative assessment of shell morphology, are needed to determine whether *R. convicta* (Clade A) and *R. perprima* (Clade D) coexist as discrete entities, as the above examples would suggest.

The reproductive relationships between the Clade D subclades are also unclear, because they do not come into contact, or they are morphologically cryptic where they do. Clade D1, restricted to the Burrup Peninsula, is genetically the most distinct, with mean COI distances of 7.2 to 10.1% from the Clades D2 to D5, and does not share morphotypes with the others. Thus Clade D1 appears to be a genetically, morphologically and geographically distinct biological species. There is less evidence for speciation among Clades D2 to D5, where there is no correspondence between morphology and the phylogenetic relationships. The phylogenetic diversity closely reflects the pattern of cyclic changes in sea level, as expected from historical fragmentation. Even on Enderby Island, where Clades D2 and D4 meet, there is geological evidence of past fragmentation during the Eemean Historical Highstand, when sea levels were 5 to 10 m higher than at present [31]. The coincidence of the phylogenetic and geographic boundaries provides some confidence that the mtDNA divergence reflects historical spatial isolation. Nevertheless, the levels of mtDNA divergence are considerably lower than among the major lineages, especially among those that neighbour or are in direct contact with one another. Although they have widths that are compatible with estimates derived from a neutral model, some of the zones of contact may be maintained by endogenous selection against recombinant genotypes, as is common for secondary hybrid zones [32,33]. More work is needed to understand how history and ecological selection have contributed to the radiation of shell form, and also their implications for speciation within the group.

## Competing interests

The authors declare that they have no competing interests.

## Authors’ contributions

SS and MSJ conceived the ideas and wrote the manuscript. SS. coordinated the fieldwork, collected the data and conducted the analyses. Both authors read and approved the final manuscript.

## Authors’ information

SS recently completed his PhD research on the evolution of *Rhagada* snails in the Dampier Archipelago, and is especially interested in the roles of history and natural selection in the evolution of morphological diversity. MSJ is emeritus professor and an honorary research fellow at the University of Western Australia, where he leads a team studying the genetics and evolution of native land snails.
